# Coumarins in the tumor-immune application: from molecular mechanisms to therapeutic innovations

**DOI:** 10.3389/fimmu.2025.1681892

**Published:** 2025-10-09

**Authors:** Yufan Zhao, Lingjie Jing, Leng Han, Cheng Guo, Quanjun Yang

**Affiliations:** Department of Pharmacy, Shanghai Sixth People’s Hospital Affiliated Shanghai Jiao Tong University School of Medicine, Shanghai, China

**Keywords:** coumarins, immunoregulation, immunotherapy, natural products, tumor microenvironment

## Abstract

Coumarin compounds represent a class of natural or synthetic molecules characterized by a benzo-α-pyrone skeleton. Their diverse substituents and extensive biological activities have garnered significant attention in the fields of anti-tumor therapy and immune regulation in recent years. This article systematically reviews the mechanisms by which coumarins influence tumor immunity. Coumarins directly inhibit tumor progression by inducing apoptosis in tumor cells, inhibiting cellular proliferation, and blocking epithelial-mesenchymal transition (EMT) and angiogenesis. However, they reshape the tumor microenvironment (TME) by regulating platelet function, macrophage polarization, T cell activity, NK cell cytotoxicity, and cytokine networks, thereby enhancing the host’s anti-tumor immune response. Despite the promising potential of coumarins in tumor immunotherapy, their mechanisms are complex, their clinical translation remains limited, and safety concerns warrant further investigation. This review summarizes the research progress on coumarins, discusses the challenges and future directions for their development, and aims to provide a comprehensive reference for the mechanism analysis and translational applications of coumarins in tumor immunotherapy. Furthermore, it seeks to facilitate the development of innovative drugs derived from natural products and to promote their clinical translation.

## 1 Introduction

Cancer remains a leading cause of death worldwide, with approximately 20 million new cases and 9.7 million deaths reported in 2022. Despite decades of conventional treatment, including surgery, chemotherapy, and radiotherapy, the prognosis for advanced-stage cancers often remains poor. This malignant disease continues to be a major problem that contemporary medicine urgently needs to address, consistently affecting the life, health, and quality of life of millions of patients (1–4). Traditional Chinese Medicine (TCM) is widely utilized in China as a complementary therapy for cancer. Research indicates that TCM herbal combinations can enhance treatment outcomes; for example, incorporating TCM into chemotherapy has been associated with improved 1-year and overall survival rates across various cancers (5). Therefore, active components from traditional Chinese medicine are a novel source with valuable anti-cancer and immunotherapy potential. These findings underscore the necessity for novel agents that are effective, affordable, and capable of synergizing with existing treatment modalities.

Although significant progress has been made in cancer immunotherapy in recent years, particularly with the advent of immune checkpoint inhibitors (ICIs) and chimeric antigen receptor T-cell (CAR-T) therapies, substantial challenges persist (6). ICIs, which target pathways such as PD-1/PD-L1 and CTLA-4, have revolutionized treatment of various cancers; however, their efficacy is frequently limited by primary or acquired resistance and can lead to severe immune-related adverse events (7, 8). Similarly, CAR-T cell therapy has demonstrated remarkable success in specific subsets of B cell leukemia and lymphoma; however, its application in solid tumors and hematological malignancies is hindered by challenges related to T cell trafficking, persistence within the immunosuppressive TME, and on-target/off-tumor toxicities (9). These limitations highlight the urgent need for complementary strategies that can modulate TME, overcome resistance mechanisms, and enhance the safety and efficacy of existing immunotherapies. Natural products, such as coumarins, which possess a multi-targeted ability to directly impact tumor cells while simultaneously reshaping the immune landscape of the TME (as detailed in subsequent sections), have emerged as compelling candidates to address this therapeutic gap and potentiate current immunotherapeutic approaches.

Coumarin compounds are widely distributed across the plant kingdom, encompassing species from the Umbelliferae, Rutaceae, and Leguminosae families as well as fungi and microorganisms. These compounds are primarily used in food, cosmetics, and medicine. Natural coumarin compounds can be extracted and separated from plants using various techniques. Structurally specific coumarin derivatives can be synthesized using chemical methods (10). Coumarin compounds are a class of natural or synthetic aromatic organic compounds characterized by a core skeleton composed of a covalently bonded benzene ring (C_6_) and an α-pyrone ring (C_5_O_2_). Owing to variations in the positions and quantities of substituents such as hydroxyl, methoxy, and isoprenyl groups, these compounds exhibit significant structural diversity, which endows them with a wide range of biological pharmacological activities, including anti-inflammatory, antioxidant, antiviral, and immunomodulatory effects. In recent years, coumarin compounds have emerged as a focal point of research in natural product medicinal chemistry, particularly for their potential in tumor prevention, treatment, and immune regulation (11–13). Based on modifications to the substituents and the expansion of the ring system, coumarin compounds can be classified into four main categories (as shown in **Table 1**): simple coumarins, furanocoumarins, pyranocoumarins, and complex coumarins. These structural variations endow coumarins with distinct pharmacological potentials (14, 15). Simple coumarins, such as 7-hydroxycoumarin and scopoletin, are prevalent in citrus fruits (lemons and grapefruits) and daisy plants (including chamomile). Their structure consists solely of a benzopyranone skeleton with a single substituent, enabling them to exert fundamental antioxidant and anti-inflammatory activities by eliminating free radicals (16, 17). Furanocoumarins, such as psoralen and xanthyletin, are characterized by a fused furan ring attached to the benzene ring, and are predominantly found in Umbelliferae plants (such as Angelica sinensis and Angelica dahurica) and Rutaceae plants (such as pepper). This class of compounds exhibits significant photosensitivity and can induce DNA cross-linking upon ultraviolet activation, making them clinically applicable in phototherapy for psoriasis (18, 19). Pyranocoumarins synthesized in select plant species from the Rutaceae and Umbelliferae families, such as praeruptorin A and clausenidin, possess a fused pyran ring that enhances molecular rigidity. These coumarin derivatives contain numerous potential anti-cancer components capable of inhibiting tumor cell proliferation and promoting apoptosis (20). Complex coumarins, including dicoumarin and the anticoagulant drug warfarin, arise from polymerization, fusion with other ring systems, or complex substitutions in the simple coumarin structure, typically exhibiting stronger biological activity and greater structural specificity (21, 22).

**Table 1:** Classification of coumarin compounds.

Class	Structure	Example
Simple Coumarins	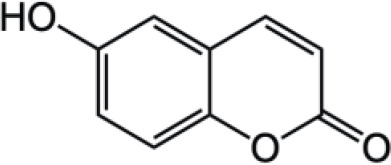	7-hydroxycoumarin
	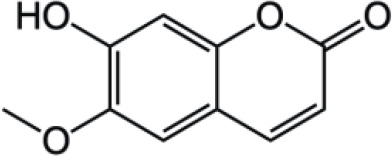	Scopoletin
Furanocoumarins	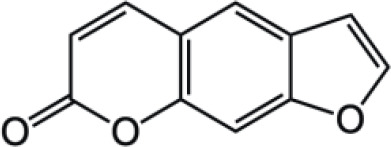	Psoralen
	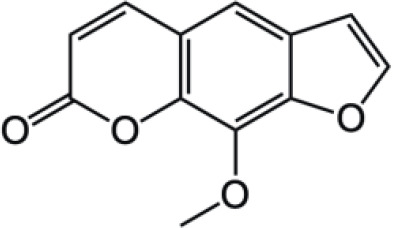	Xanthotoxin
Pyranocoumarins	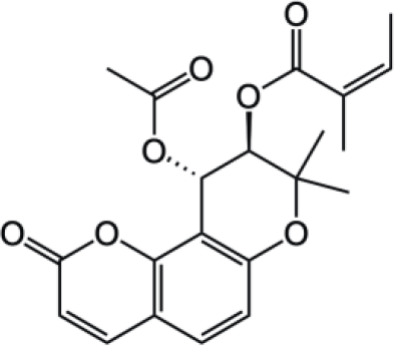	Praeruptorin A
	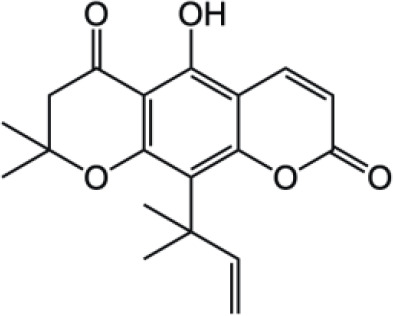	Clausenidin
Complex Coumarins	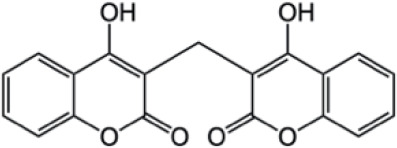	Dicoumarin

Coumarins, an ancient and diverse family of natural products, possess an α-phenyldipyranone framework. This framework, which is characterized by a variety of substituents and differing ring fusion patterns, has resulted in a wide array of types and structures. Their extensive natural sources and straightforward biosynthetic pathways provide opportunities for metabolic engineering and chemical modification. Additionally, their rich pharmacological properties provide a solid foundation for applications across multiple fields, including anticoagulation and anti-inflammatory, neuroprotective, and antibacterial activities. In the subsequent chapters, we will explore in depth the specific mechanisms of action of coumarins in anti-tumor and immune regulation, the advancements in preclinical research, and the potential for clinical applications. Furthermore, we will conduct a thorough analysis of the safety challenges encountered and outline future research directions, with the aim of promoting innovative applications of coumarins in tumor immunotherapy.

## 2 Molecular mechanism of anti-tumor effects of coumarins

**Figure 1:**
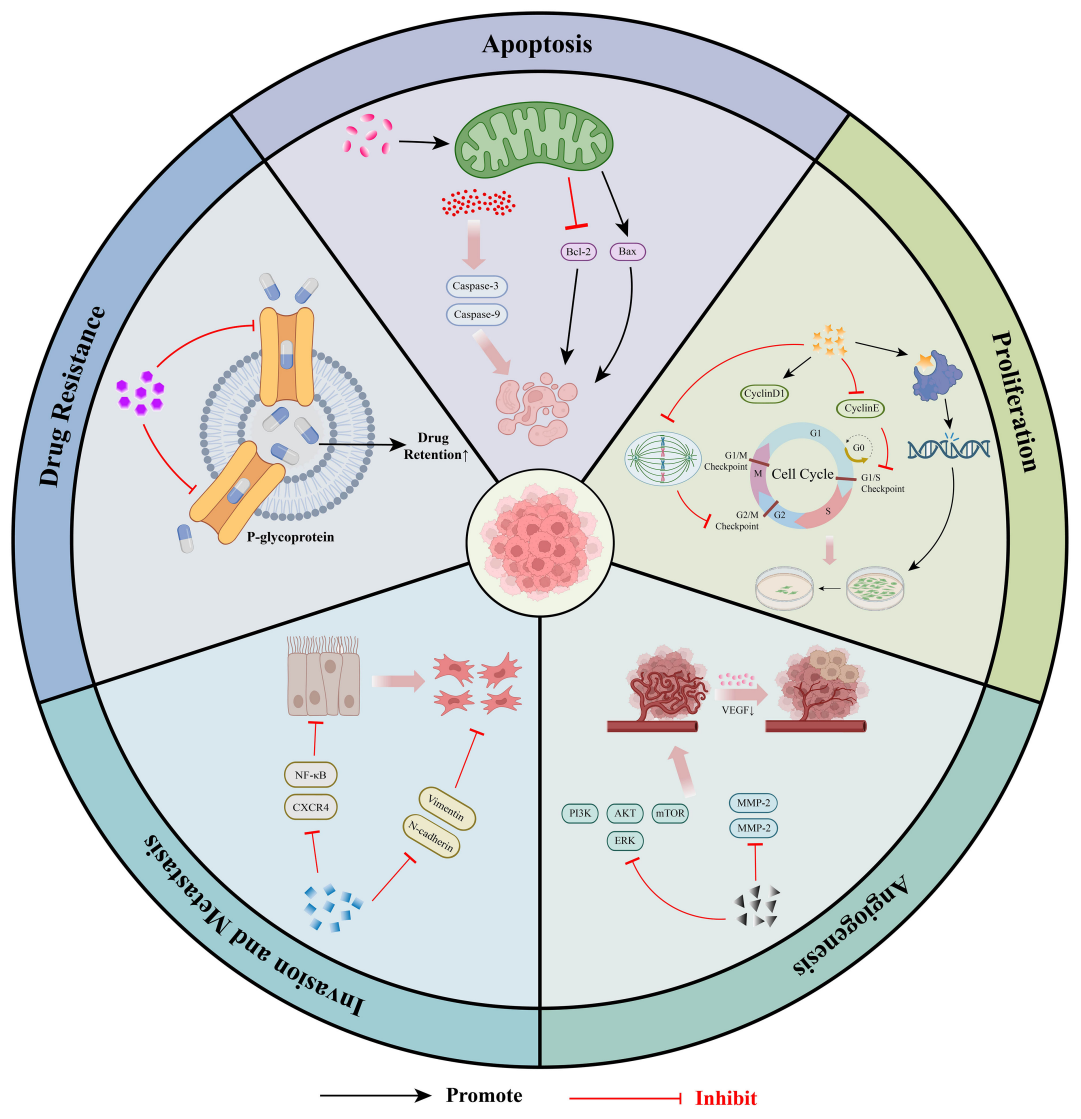
Molecular mechanism of anti-tumor effects of coumarins: ① Coumarins can induce cancer cell apoptosis by activating pro-apoptotic signaling pathways and regulating mitochondrial function. ②Coumarins can inhibit the proliferation of tumor cells by blocking the cell cycle process, interfering with DNA synthesis and disrupting the microtubule structure. ③Coumarins can inhibit tumor angiogenesis by down-regulating the expression of VEGF and MMPs. ④Coumarins can inhibit the invasion and metastasis of tumors by regulating EMT and blocking the relevant signaling pathways related to metastasis.⑤Coumarins can enhance the accumulation of chemotherapeutic drugs within tumor cells by regulating the expression of drug resistance-related genes and proteins, effectively reversing MDR.

Studies have demonstrated that Coumarins and their derivatives exhibit remarkable anti-cancer, anti-tumor, and anti-proliferative activities. Coumarin compounds are not only effective anti-cancer agents but also exhibit minimal side effects (23). These bioactive substances display significant anti-cancer activities across various substitution patterns. In the subsequent sections, we focus on introducing five key mechanisms underlying the anti-tumor potential of coumarins (**Figure 1**).

### 2.1 The effect of coumarins on cancer cell apoptosis

Coumarin compounds can induce apoptosis in cancer cells through multiple pathways, primarily by regulating apoptosis-related signaling pathways and expression of key proteins. Research has demonstrated that Coumarins not only activate pro-apoptotic signaling pathways but also modulate mitochondrial function, thereby exerting anti-tumor effects in various cancer models.

At the molecular level, coumarins significantly enhance the activity of extracellular c-Jun N-terminal kinase (JNK) and p38 mitogen-activated protein kinase (MAPK) signaling pathways, which increases the sensitivity of cancer cells to apoptotic signals (24). Further investigations have revealed that coumarins exert their effects through both the endogenous and exogenous apoptotic pathways. In the endogenous apoptotic pathway, coumarins act on the mitochondria, inducing the release of pro-apoptotic factors, which activate apoptotic proteases such as caspase-9 and caspase-3, upregulate the pro-apoptotic protein Bax, and downregulate the anti-apoptotic protein Bcl-2. This cascade results in a decrease in the mitochondrial membrane potential, release of cytochrome c, and initiation of the apoptotic signaling cascade (25). Moreover, experimental evidence indicates that coumarins can significantly induce apoptosis in various cancer cell lines, including those of breast and lung cancers, suggesting their potential for broad-spectrum anti-cancer applications (26).

In conclusion, coumarin compounds effectively promote apoptosis in cancer cells through multi-target and multi-pathway synergies. Their unique molecular mechanisms not only provide new insights for the development of anti-tumor drugs but also establish a vital foundation for the in-depth exploration of the anti-cancer activities of natural products.

### 2.2 The effect of coumarins on cancer cell proliferation

Coumarin compounds inhibit tumor cell proliferation through multiple targets and pathways, primarily by blocking cell cycle progression, interfering with DNA synthesis, and disrupting the microtubule structure. These effects enable coumarins to exhibit significant anti-proliferative activity in various cancer models, providing crucial insights for the development of anti-tumor drugs.

Coumarins can regulate the expression of key cyclins, leading to cell cycle arrest in tumor cells at various stages, particularly at the G1 or G2/M phases. For instance, psoralen upregulates Cyclin D1 and downregulates Cyclin E1 expression during the G1 phase, with Cyclin E1 serving as a critical regulator of the cell cycle. The alteration of cyclin levels inhibits the transition of cells from G1 to S phase, thereby impeding cell division and proliferation (27). Additionally, 4-substituted coumarin derivatives (such as SKLB060) act as novel tubulin-targeting agents, binding to the colchicine site on tubulin and disrupting microtubule polymerization. This action results in microtubule depolymerization, which interferes with the normal function of the mitotic spindle during cell division, ultimately leading to cell cycle arrest in the G2/M phase (28). Some coumarin derivatives (such as S009-131, a coumarin-chalcone hybrid) can directly induce DNA damage by intercalating into the minor groove of the DNA, thereby disrupting normal cellular processes. Given that the DNA repair capacity of tumor cells is typically compromised, such damage further inhibits their proliferation (29). Furthermore, the coumarin analogue AD-013 has been shown to inhibit the activity of the Breast Cancer Gene 1 (BRCA1) protein, reduce the expression of DNA-dependent protein kinase (DNA-PK), and simultaneously activate Ataxia-telangiectasia mutated (ATM) and Ataxia telangiectasia and Rad3-related (ATR) proteins, as well as p53. This cascade of events leads to the accumulation of DNA breaks and ultimately results in cell death (30).

In conclusion, coumarin compounds effectively inhibited tumor cell proliferation through multiple mechanisms, including cell cycle regulation, disruption of microtubule dynamics, and induction of DNA damage. These findings not only elucidate the molecular mechanisms underlying the action of coumarins but also provide a theoretical foundation for the development of new anti-tumor drugs.

### 2.3 The effect of coumarins on cancer angiogenesis

Tumor growth and metastasis are significantly reliant on the supply of nutrients and oxygen facilitated by newly formed blood vessels. Coumarin compounds have emerged as a focal point in anti-tumor research because of their distinctive anti-angiogenic properties. Numerous studies have demonstrated that coumarin can inhibit tumor angiogenesis through various targets and pathways, effectively disrupting the nutrient supply and routes of metastasis in tumors, thereby showing promising potential for anti-tumor applications.

Coumarin directly interacts with vascular endothelial cells and obstructs the formation of new blood vessels by inhibiting their proliferation and migration. Studies have indicated that coumarin can significantly downregulate the expression of vascular endothelial growth factor (VEGF), a crucial regulatory factor in angiogenesis (31). At the molecular level, coumarin inhibits key signaling pathways such as PI3K/Akt/mTOR and ERK. This inhibition not only diminishes the secretion of angiogenesis-related factors but also directly impairs the nutrient acquisition capability of tumor cells, effectively curtailing blood vessel formation and further restricting tumor growth and dissemination (32, 33). Additionally, coumarin compounds can effectively inhibit degradation of the extracellular matrix by specifically downregulating the expression of matrix metalloproteinases (MMPs), particularly MMP-2 and MMP-9. This effect directly impedes the sprouting and extension of tumor blood vessels, offering a novel intervention target for limiting tumor metastasis (34).

Current research has elucidated the molecular mechanisms underlying the anti-angiogenic effects of coumarin. However, the precise regulatory network governing its multi-target synergistic action requires further investigation. Future studies should prioritize optimizing the structure of coumarin derivatives to enhance their targeting capabilities, thereby providing a critical theoretical foundation and clinical pathway for the development of new anti-angiogenic drugs.

### 2.4 The effect of coumarins on cancer cell invasion and metastasis

Tumor invasion and metastasis are the primary causes of mortality in cancer patients. The metastasis process is selective; only those cells that can successfully invade, embolize, survive in circulation, remain in the distant capillary bed, and proliferate within the organ parenchyma can form metastases (35). Recent studies have demonstrated that coumarin compounds significantly inhibit tumor invasion and metastasis through multi-target and multi-pathway mechanisms. Their mechanisms of action primarily include regulation of EMT, inhibition of extracellular matrix degradation, and blockade of metastasis-related signaling pathways, indicating promising prospects for the development of anti-metastatic drugs (36, 37).

EMT is a reversible biological process wherein tumor cells lose their epithelial phenotype and acquire a mesenchymal phenotype, serving as a crucial mechanism for tumor invasion and metastasis (38). Auraptene, a natural coumarin, inhibits the migration and invasion of melanoma cells by regulating EMT markers and reducing the activity of MMP-2 and MMP-9. During the EMT process, upregulation of N-cadherin expression allows cancer cells to detach from neighboring cells and gain invasive capabilities. Vimentin provides structural support for cell migration and invasion. Auraptene prevents cell migration and invasion by downregulating N-cadherin and vimentin expression Furthermore, Auraptene reduces the activity of matrix metalloproteinases (MMPs), particularly MMP-2 and MMP-9, effectively inhibiting degradation of the extracellular matrix (ECM) (39). At the signaling pathway level, isoimperatorin (IIPT), a natural furanocoumarin, can inhibit EMT in colorectal and liver cancer cells by suppressing the activation of the NF-κB signaling pathway and the expression of CXCR4. CXCR4 overexpression has been reported to be associated with metastasis in various cancers (40).

These studies indicate that coumarin compounds exert their anti-tumor effects not only through traditional mechanisms such as apoptosis, but also by limiting the metastatic potential of tumors by targeting the EMT process. This further supports their potential as anti-tumor drugs. Despite extensive preclinical research confirming the anti-metastatic activity of coumarins, challenges remain in their clinical application. Future research should prioritize the development of coumarin derivatives that specifically target the key regulatory factors of EMT.

### 2.5 The effect of coumarins on cancer drug resistance

In recent years, coumarin compounds, as natural products, have demonstrated unique potential in reversing multidrug resistance (MDR) in tumors. Drug resistance is the primary cause of chemotherapy failure, and its mechanisms often involve overexpression of drug efflux pumps (such as P-glycoprotein) and inhibition of apoptotic signaling pathways (41). Studies have shown that coumarin derivatives can regulate the expression of drug resistance-related genes and proteins through multi-target effects, enhance the accumulation of chemotherapeutic drugs in tumor cells, and effectively reverse MDR.

In the calcein-AM uptake assay of MDCK-MDR1 cells, a series of conjugates linked to the 1,2,3,4-tetrahydroisoquinoline motif and substituted 7-hydroxy-2H-chromen-2-ones (coumarin derivatives) was tested. The results indicated that these compounds could inhibit the function of P-gp. When used in combination with the well-known anti-cancer drug doxorubicin, some coumarin-containing compounds (such as 3d, 3h, 3l, 3r, 3t, and 3u) reversed MDR by inhibiting P-glycoprotein-mediated drug efflux, thereby restoring the anti-proliferative effect of doxorubicin on drug-resistant cells. These compounds exhibited dose-dependent enhancement of doxorubicin (10 μM) cytotoxicity (42). Enhancing chemotherapeutic retention in tumors represents a promising application for coumarins. Certain coumarin derivatives have demonstrated the ability to inhibit key efflux pumps, such as P-gp and breast cancer resistance protein (BCRP), significantly increasing the intracellular levels of drugs like paclitaxel. This mechanism has the potential to improve the efficacy of standard chemotherapeutic agents in resistant cancers (43). The coumarin compound auraptenol triggers apoptosis in drug-resistant prostate cancer cells by inducing reactive oxygen species (ROS). Auraptenol inhibited p38 phosphorylation in a dose-dependent manner, and the expression of JNK was concentration-dependent. It blocked the JNK/p38 MAPK signaling pathway in a concentration-dependent manner in human prostate cancer cells, indicating its potential as an anti-cancer agent (44).

Coumarin compounds represent a novel strategy for reversing tumor MDR by targeting drug resistance genes and regulating apoptotic pathways. Future research should focus on further optimizing their pharmacokinetic properties (such as extending half-life) and exploring combination treatment regimens to address the dynamic evolution of tumor resistance mechanisms.

It is important to recognize that the direct anti-tumor effects of coumarins are not isolated. Critically, these actions can synergize with and enhance immunomodulatory functions within the TME. For instance, coumarins may directly modulate immune cell functions while concurrently exerting direct anti-tumor actions. This intricate interplay between direct cytotoxicity and immune reprogramming underscores the multifaceted potential of coumarins in comprehensive tumor control, which will be elaborated in the following section, focusing on their specific immunoregulatory mechanisms.

## 3 Regulatory mechanisms of coumarins in tumor immunity

**Figure 2:**
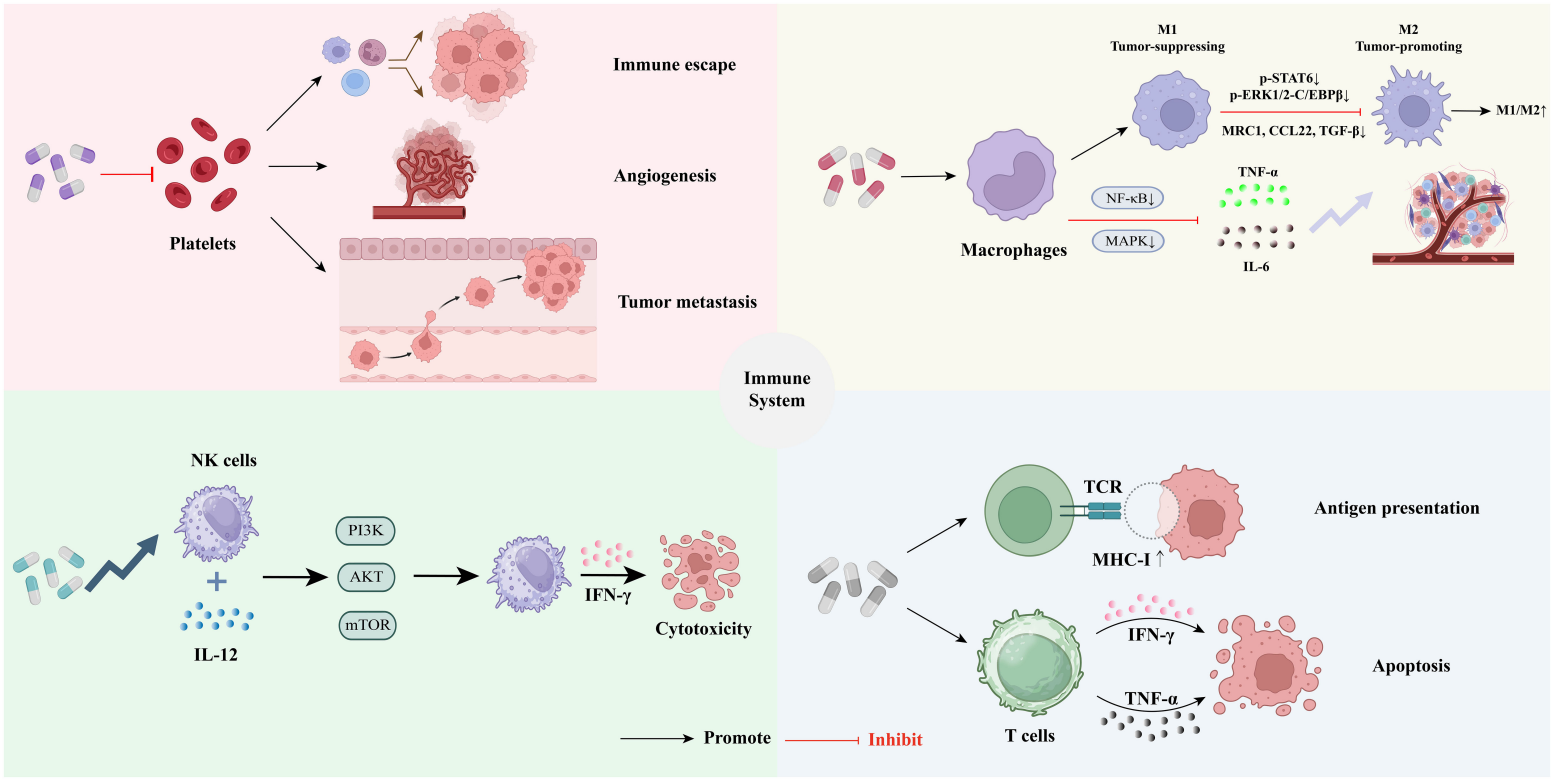
Regulatory mechanisms of coumarins in tumor immunity: ① Coumarin have the potential to regulate tumor-associated platelet functions ②Coumarin can inhibit chronic inflammation and reshape the tumor immune microenvironment by regulating the functions of macrophages. ③Coumarins can enhance the function of natural killer cells, thereby exerting anti-tumor effects. ④Coumarins can enhance the effector function of T cells to strengthen the tumor immune response.

The TME constitutes a complex ecosystem characterized by dynamic interactions among immune cells, stromal components, and tumor cells, often leading to immunosuppression and tumor progression (45). Cold tumors are characterized by low immune infiltration, exhibiting immune-excluded phenotypes. These tumors generally demonstrate a poor response to immunotherapy. To effectively address the challenges posed by cold tumor microenvironments, it is often necessary to implement strategies that either stimulate immune cell infiltration or mitigate local immunosuppression (46). Coumarins, owing to their multi-target immunomodulatory properties, can disrupt this immunosuppressive network by directly or indirectly influencing key immune cell populations (45, 47). The subsequent sections systematically examined how coumarins reshape the TME by targeting platelets, macrophages, Natural killer (NK) cells, T cells, and B cells, ultimately restoring anti-tumor immunity. Importantly, these immunomodulatory effects are not isolated; rather, they are interconnected and collectively contribute to a more favorable immune landscape for tumor control (**Figure 2**).

### 3.1 Platelets

The normal platelet range is approximately 150,000 to 450,000 cells/µL of blood. However, many cancer patients exhibit thrombocytosis (elevated platelet levels), which is associated with poorer clinical outcomes (48, 49). Platelets are not only key players in hemostasis and thrombosis but also play significant roles in inflammatory responses and immune regulation. They promote the activation and migration of immune cells by releasing cytokines, chemokines, and microparticles. Additionally, platelets mediate the recruitment and activation of immune cells through their surface receptors (50–52).

In recent years, the regulatory effects of coumarin compounds on platelet function have attracted increasing attention. These compounds participate in the antithrombotic process through anticoagulation and inhibition of platelet aggregation while also regulating the signaling pathways of platelet activation, thereby influencing their immune functions (53, 54). This mechanism offers new strategies for the therapeutic application of coumarin drugs in thrombotic- and inflammatory-related diseases.

It is worth noting that platelets also play a significant role in cancer progression, including promoting immune escape of tumor cells, enhancing angiogenesis, and facilitating tumor metastasis. Platelets shield these cells from immune system attacks and promote their adhesion to the vascular endothelium, thereby increasing their metastatic potential (55). This raises a crucial question: Can coumarin compounds exert anti-tumor effects by modulating the platelet-tumor axis? For example, coumarins can inhibit platelet activation and aggregation induced by tumor cells, thereby diminishing their metastatic potential. Furthermore, the antithrombotic and anti-inflammatory effects of coumarins can effectively impede tumor-related angiogenesis and formation of inflammatory microenvironments. These questions necessitate further mechanistic studies and experimental validations.

Although coumarin compounds have the potential to regulate tumor-associated platelet functions, their clinical application remains challenging. Their anticoagulant properties may increase the risk of bleeding, with significant individual variability. Thus, systematic clinical research is urgently required to assess their efficacy and safety and to establish a foundation for their application in cancer treatment.

### 3.2 Monocytes/macrophages

Monocytes and macrophages are crucial components of the innate immune system and are primarily responsible for defending against external invasion through phagocytosis of pathogens, secretion of cytokines, and mediation of inflammatory responses (56, 57). In the TME, these cells exhibit ‘double-edged sword’ characteristics. Monocytes and tumor-associated macrophages (TAMs) can promote tumor growth and development by secreting pro-inflammatory factors, facilitating angiogenesis, and suppressing anti-tumor immunity; however, they can also exert anti-tumor effects under specific polarization states, particularly when polarized towards the M1 type (58, 59).

Research indicates that coumarin compounds can mitigate local or systemic inflammatory responses by inhibiting key inflammatory signaling pathways, such as NF-κB and MAPK, thereby reducing the secretion of proinflammatory cytokines by macrophages, including TNF-α and IL-6. This action is expected to alleviate the tumor-related inflammatory microenvironment (60). For example, 4-methylumbelliferone (4-MU) primarily exerts its anti-tumor effects by regulating inflammatory factors, particularly through the inhibition of IL-6 production and angiogenesis. IL-6 is a pivotal factor that drives the activation of the JAK/STAT3 signaling pathway, which is critical for promoting tumor angiogenesis and immune evasion (61).

Furthermore, coumarin compounds can regulate the polarization state of macrophages, inhibit their transformation into the pro-tumorigenic M2 type, and increase the M1/M2 ratio, thereby reshaping the immune landscape of the TME. M1-type macrophages exhibit significant anti-tumor functions, whereas M2-type macrophages are closely associated with tumor growth, immune suppression, and angiogenesis (62). Wang et al. further elucidated the underlying mechanism, indicating that osthole can block the M2 polarization process by inhibiting the p-STAT6 and p-ERK1/2-C/EBPβ signaling axis, which significantly reduces the expression of M2 markers, such as MRC1, CCL22, and TGF-β, thereby interfering with the formation of tumor-promoting macrophages (63).

This shift in macrophage polarization and the cytokine profile induced by coumarins creates a more favorable immunological milieu within the TME. Notably, the reduction of immunosuppressive cytokines, such as TGF-β, in conjunction with potential increases in immunostimulatory signals, can significantly enhance the activity and proliferation of CD8+ cytotoxic T lymphocytes (discussed in Section 3.4) and augment the cytotoxicity of Natural Killer (NK) cells (discussed in Section 3.3), thereby fostering a more robust anti-tumor immune response.

In conclusion, coumarin compounds may play a crucial role in inhibiting chronic inflammation and reshaping the tumor immune microenvironment by regulating the functions of monocytes and macrophages. They have the potential to enhance anti-tumor immune responses and may serve as an adjunctive means of immunotherapy to alleviate treatment-related inflammatory responses, possessing significant clinical translational value.

### 3.3 Natural killer cells

NK cells are a vital subtype of innate lymphoid cells (ILCs) that play a crucial role in the body’s innate immune defense, particularly in anti-tumor immunity. NK cells can recognize and directly eliminate virus-infected and malignant tumor cells, exhibiting significant cytotoxic activity while secreting various cytokines and chemokines including interferon-γ (IFN-γ). They are predominantly found in the peripheral blood, spleen, and bone marrow and serve as key effector cells in tumor immune surveillance and clearance. Given the increasing focus on tumor immunotherapy, NK cells have garnered widespread research interest as potential therapeutic targets (64, 65).

Daphnetin, a natural coumarin compound, has been shown to significantly enhance NK cell function, particularly in the context of anti-tumor immunity. Research indicates that daphnetin can synergize with IL-12 to boost NK cell function by activating the PI3K-Akt-mTOR signaling pathway, which promotes IFN-γ secretion and increases cytotoxicity in tumor cells (66). Furthermore, Han et al. demonstrated that the coumarin derivative nodakenin can enhance NK cell activity and cytokine production capacity, highlighting its potential to augment anti-tumor immune responses in immunocompromised states and providing a theoretical foundation for its consideration as a candidate drug in tumor immunotherapy (67).

Although current research on the regulation of NK cell anti-tumor functions by coumarin compounds is relatively limited, this field presents significant research prospects. Future studies should investigate whether coumarin and its derivatives can enhance the recognition and cytotoxicity of NK cells against tumor cells by modulating the expression of activating receptors on NK cell surface. Additionally, research should explore the potential of coumarins to promote NK cell-mediated immune surveillance by influencing the expression of ligands on tumor cell surfaces (68). Notably, the TME encompasses various immunosuppressive factors, such as TGF-β, which can significantly inhibit NK cell activity. Preliminary studies indicate that certain coumarins, such as esculetin, may counteract tumor-induced NK cell dysfunction by inhibiting the TGF-β signaling pathway, thereby suppressing tumor cell proliferation, inducing apoptosis, and enhancing immune responses (69–71).

In conclusion, coumarin compounds have demonstrated pharmacological activities in enhancing NK cell function and anti-tumor potential. However, further mechanistic research and preclinical validation are urgently required to broaden their application in tumor immunotherapy.

### 3.4 T cells

T cells are fundamental components of the adaptive immune system and play a crucial role in tumor immune surveillance and elimination (72). Among these, CD8+ cytotoxic T lymphocytes (CTLs) directly induce tumor cell apoptosis by secreting perforin and granzyme (73). In contrast, CD4+ helper T cells facilitate the activation, proliferation, and sustained effector function of CD8+ T cells by providing essential stimulatory signals and cytokines, thereby enhancing the overall anti-tumor immune response (74). Furthermore, regulatory T cells (Tregs) are typically immunosuppressive within the TME, impeding the host’s anti-tumor immune response by secreting inhibitory cytokines such as IL-10, TGF-β, and IL-35, and have emerged as significant therapeutic targets in contemporary anti-tumor immune interventions (75).

Urolithin A (UA) is a metabolite of benzo coumarin that regulates T-cell function across multiple dimensions. Denk et al. demonstrated that UA promotes the generation of T memory stem cells (TSCM) with robust anti-tumor capabilities and enhances antigen presentation efficiency by upregulating MHC-I molecule expression on the surface of tumor cells. This action reduces T cell exhaustion and significantly boosts the anti-tumor immune response (76). Additionally, Ma et al. indicated that UA enhances the metabolic adaptation and persistence of CD8+ T cells by activating the ERK1/2-ULK1 signaling pathway, thereby improving their effector function and potentially augmenting the long-term efficacy of immunotherapy (77).

Wedelolactone, a naturally occurring coumarin, enhances IFN-γ signal transduction by inhibiting the phosphatase activity of T-cell protein tyrosine phosphatase (TCPTP). This inhibition prolongs tyrosine phosphorylation of STAT1, a key signaling molecule in the IFN-γ pathway, thereby promoting tumor cell apoptosis in a STAT1-dependent manner (78). Another coumarin compound, umbelliprenin, exerts its anti-tumor effect by regulating the immune response through the promotion of pro-inflammatory cytokines such as IFN-γ and TNF-α. IFN-γ plays a crucial role in enhancing tumor immune responses by increasing T-cell activity to target and eliminate cancer cells. TNF-α is well known for its ability to induce apoptosis in cancer cells and promote inflammation (79).

Notably, T-cell function is often significantly suppressed during tumor immune escape (80). Coumarin compounds, as potential immunomodulators, are anticipated to disrupt the immunosuppressive state by restoring or enhancing T-cell effector functions, thereby inhibiting tumor growth and delaying progression. Furthermore, there may be a synergistic effect between coumarins and T cell-related immunotherapy strategies, which is expected to further broaden their application prospects in the treatment of solid tumors (47).

### 3.5 B cells

B cells are a crucial component of the immune system, primarily responsible for antibody production and the formation of immune memory (81). In the context of tumor immunity, the role of B cells is complex and varies depending on their subtype and functional state. B cells can enhance immune surveillance by generating anti-tumor antibodies or, conversely, facilitate tumor progression within the TME by secreting immunosuppressive factors or producing oncogenic antibody-antigen complexes (82). Therefore, further investigation into the mechanisms of B cell action and regulation across different tumors is essential for the advancement of more effective anti-tumor immunotherapies.

Although existing literature on the regulatory effects of coumarins on B cells is relatively sparse, we can still reference previous studies to propose viewpoints that may stimulate future research. For example, coumarins and their derivatives may modulate B cell activation and proliferation, inhibit the release of certain cytokines (such as IL-6 and TNF-α), reduce the generation and activation of regulatory B cells (Bregs), and diminish their immunosuppressive effects on T cells, thereby enhancing the anti-tumor immune response (82, 83). Additionally, it is important to consider the synergistic effects on anti-tumor immune responses. In various tumor immunotherapy models, coumarin compounds have demonstrated synergistic effects when combined with other immunotherapy strategies (such as immune checkpoint inhibitors). By modulating B cell functions, coumarins may enhance the production of anti-tumor antibodies, strengthen the collaboration between T and B cells, and further promote the anti-tumor immune response (82, 84).

Although coumarin has the potential to regulate B-cell immunity, its effects may vary among individuals. Different types of tumors may exhibit distinct immune escape mechanisms; therefore, the regulatory effect of coumarin on B cells may differ among various tumor types. Future research should focus on the precise regulation of B cell immune function by coumarin, explore its synergistic effects with other immunotherapeutic approaches, and further evaluate its safety and efficacy in clinical treatment.

## 4 Discussion

### 4.1 Comprehensive treatment of coumarins

Coumarin compounds can enhance the overall therapeutic efficacy of tumors via synergistic effects in chemotherapy, radiotherapy, and immunotherapy. The mechanism lies in its ability to reverse drug resistance and reshape the TME. For instance, 5-fluorouracil is the most widely used drug for treating colorectal cancer; however, its efficacy is often limited by drug resistance. The coumarin derivative esculetin has been shown to inhibit the proliferation, migration, and EMT of colorectal cancer cells. When used in combination with 5-fluorouracil, esculetin enhances the inhibitory effects of these processes, thereby improving therapeutic outcomes (85). Additionally, coumarin compounds can mitigate the side effects associated with radiotherapy, demonstrating promising results in cancer treatment. This indicates their potential to alleviate adverse effects frequently experienced by patients during radiotherapy, such as damage to healthy tissues and cells. Furthermore, Mahler et al. indicated that the combination of coumarin and triciribine positively affects the treatment of malignant tumors in head and neck radiotherapy (26).

Beyond traditional chemotherapy and radiotherapy, emerging evidence has highlighted the synergistic potential of coumarins in conjunction with novel immunotherapeutic strategies. For instance, the combination of trametinib (a MEK inhibitor) and 4-methylumbelliferone (4-MU) has been shown to significantly downregulates the expression of programmed cell death protein 1 (PD-1) and its ligand (PD-L1). This suggests that coumarins such as 4-MU may sensitize tumors to immune checkpoint blockade by alleviating the immunosuppressive TME, potentially overcoming resistance, and reducing the required doses of checkpoint inhibitors, thereby mitigating their associated toxicities (86). GLP-1 and Urolithin A enhance CAR T cell persistence and anti-tumor activity by targeting autophagy and mitochondrial metabolism. Akhtar et al. discovered that Urolithin A directly enhances mitophagy, synergizes with GLP-1 to eliminate dysfunctional mitochondria, and improves oxidative phosphorylation. Collectively, these agents restore metabolic balance by shifting CAR T cells from glycolysis-driven exhaustion to mitochondrial respiration, thereby supporting memory T-cell generation and persistence (87). Based on the previously mentioned ability of coumarins to regulate key immune cells within the TME, there is a robust theoretical basis for their combination with adoptive cell therapies, such as CAR-T cells. By reshaping the TME to be less immunosuppressive and more conducive to immune cell activity, coumarins could potentially enhance the infiltration, persistence, and efficacy of CAR-T cells, particularly in solid tumors, where the TME poses a significant barrier. Exploring these combinations represents a promising direction for future therapeutic development by leveraging the immunomodulatory properties of coumarins to augment the efficacy of cutting-edge immunotherapies.

### 4.2 Challenges of scientific mechanism

Coumarin compounds exhibit multitarget characteristics and can influence various signaling pathways, including RAF/MEK/ERK, PI3K/AKT, STAT3, NF-κB, and PD-1/PD-L1. While this multi-target capability provides a broad foundation for anti-tumor immune effects, it also complicates the investigation of the underlying mechanisms. For example, the predominant mechanism remains unclear, complicating the identification of the targets or pathways primarily responsible for anti-tumor immune responses. Furthermore, interactions between different pathways can lead to signal network cross-interference, resulting in unpredictable biological effects. Additionally, there is a scarcity of direct studies examining the dual role of coumarins in immune regulation. Specific experiments are required to verify and elucidate the specific microenvironments that may exhibit the immunosuppressive effects of coumarins. Investigating whether significant differences exist in the responses of various tumor types or subtypes to coumarins represents another promising area for future research.

### 4.3 Clinical transformation and safety challenges

Despite compelling preclinical evidence supporting the anti-tumor and immunomodulatory activities of coumarins, their clinical translation faces substantial hurdles. Most research remains firmly rooted in the preclinical stage and relies heavily on *in vitro* cell culture models and murine studies. Although these models provide valuable mechanistic insights, they often fail to fully recapitulate the complexity of human tumors and the human immune system. Consequently, clinical trial data evaluating coumarins specifically for tumor immunotherapy in humans are extremely scarce and preliminary. A critical barrier is the limited understanding of the pharmacokinetic (PK) and pharmacodynamic (PD) profiles of coumarin derivatives in humans. Issues such as poor aqueous solubility, low oral bioavailability, rapid metabolism (often mediated by cytochrome P450 enzymes), and short half-life significantly hamper their delivery to the tumor site and sustained therapeutic effects (17, 88). For instance, promising compounds such as dicoumarol exhibit favorable activity *in vitro*, but suffer from rapid clearance and unpredictable PK *in vivo*, limiting their clinical utility (89). Furthermore, safety concerns associated with long-term administration, particularly hepatotoxicity (as seen with some furanocoumarins such as imperatorin) and phototoxicity (characteristic of linear furanocoumarins like psoralen), pose significant clinical risks and necessitate careful dose optimization and patient selection (90, 91). Even in the context of combination therapies, the optimal dosing schedules, potential drug-drug interactions, and long-term safety profiles of coumarins paired with standard therapies or immunotherapies remain largely unexplored in human trials. Addressing PK/PD limitations, along with safety challenges, through structural optimization strategies (such as prodrug strategies), advanced delivery systems (including nanoparticles and liposomes), and rigorous Phase I clinical trials are essential for realizing the full therapeutic potential of coumarins in tumor immunotherapy in clinical settings.

## 5 Conclusion

Coumarin derivatives, which are characterized by their unique molecular structures and diverse biological activities, have demonstrated significant potential in tumor immunotherapy. Research indicates that coumarins not only exert direct effects on tumor cells to inhibit proliferation and induce apoptosis but also enhance anti-tumor immune responses by modulating the TME. Their mechanisms of action involve multiple signaling pathways, including NF-κB, STAT3, and PI3K/Akt/mTOR, which are crucial for regulating immune cell activity, inflammatory responses, angiogenesis, and immune evasion.

First, coumarin derivatives directly inhibit tumor cell growth and survival through various mechanisms such as inducing mitochondrial apoptosis pathways, blocking cell cycle progression, interfering with DNA synthesis, and inhibiting microtubule polymerization. These mechanisms exhibit strong anti-tumor effects across various cancer types, particularly in breast, lung, and colorectal cancers. The multi-target nature of coumarins allows them to effectively counteract the resistance of cancer cells to conventional treatments such as chemotherapy and radiotherapy, thereby enhancing treatment efficacy. Furthermore, coumarins can inhibit the EMT which diminishes the migratory and invasive capabilities of tumor cells, consequently reducing the risk of metastasis.

Second, coumarins play a crucial role in tumor immune regulation. Studies have demonstrated that coumarins can modulate the activity of immune cells within the TME, thereby enhancing anti-tumor immune responses. For instance, coumarins can influence the polarization of macrophages, inhibit the immunosuppressive effects of tumor-associated macrophages (TAMs), and promote the anti-tumor activity of M1-type macrophages. Additionally, coumarins can regulate the activation state of T cells, augment the cytotoxic effects of CD8+ T cells, and mitigate the suppression of the immune system by regulatory T cells (Tregs), thereby bolstering anti-tumor immune responses. Furthermore, coumarins exhibit the potential to enhance natural killer (NK) cell activity and promote B cell-mediated antibody-dependent cell cytotoxicity (ADCC), thereby expanding their applicability in tumor immunotherapy.

Despite the numerous advantages of coumarins in anti-tumor immunity, their clinical translation faces several challenges. First, the multi-target effects of coumarins, while providing extensive pharmacological activities, complicate research efforts. Currently, the mechanisms of action of coumarins in immune regulation remain incompletely understood, particularly across different tumor types, where the primary targets and molecular mechanisms may vary, necessitating further systematic investigation. Second, clinical trial data on coumarins are still limited. Although some *in vitro* experiments and animal model studies have suggested their anti-tumor potential, further evaluation of their pharmacokinetic characteristics, safety, and efficacy in humans is required. Moreover, the potential side effects of coumarin derivatives, such as phototoxicity and hepatotoxicity, may hinder their clinical application, necessitating structural optimization and dose adjustments to mitigate adverse reactions.

Future research should focus on the following aspects. First, a more detailed elucidation of the molecular mechanisms of coumarins is essential to determine optimal application strategies for different tumor types. Second, it is imperative to enhance research on the combined application of coumarins with other therapeutic modalities (such as immune checkpoint inhibitors, CAR-T cell therapy, and radiotherapy) to improve their anti-tumor efficacy and mitigate drug resistance. Furthermore, the development of structural modifications and nanodelivery systems presents new avenues for the clinical application of coumarins. By optimizing their pharmacological properties and enhancing targeting and bioavailability, more precise and effective anti-tumor treatments can be achieved.

In conclusion, coumarin-based compounds have demonstrated multifaceted potential in anti-tumor immunotherapy. They modulate the TME through multi-target mechanisms and bolster anti-tumor immune responses, thus providing a novel direction for anti-tumor treatment. However, their successful translation into clinical practice hinges on thorough exploration of the complexities of their mechanisms, safety concerns, and clinical indications. Through interdisciplinary collaboration and technological advancements, coumarins are poised to become significant adjuncts to tumor immunotherapy, offering safer and more effective treatment options for patients. Future research must continuously address three critical areas: depth of mechanism understanding, clinical validation, and technological innovation to facilitate the transition of this natural molecule from laboratory settings to clinical applications.
